# Fluoroquinolones for the Prophylaxis of Spontaneous Bacterial Peritonitis in Patients with Liver Cirrhosis: Are They Losing Ground?

**DOI:** 10.3390/life15040586

**Published:** 2025-04-02

**Authors:** Simona Juncu, Horia Minea, Andreea Lungu, Alina Jucan, Raluca Avram, Ana-Maria Buzuleac, Camelia Cojocariu, Laura Sorina Diaconu, Carol Stanciu, Anca Trifan, Ana-Maria Sîngeap

**Affiliations:** 1 Department of Gastroenterology, Faculty of Medicine, “Grigore T. Popa” University of Medicine and Pharmacy, Universitatii Street No. 16, 700115 Iasi, Romania; juncu_simona-stefania@d.umfiasi.ro (S.J.); zvincu.andreea@d.umfiasi.ro (A.L.); ghiata.alina-ecaterina@d.umfiasi.ro (A.J.); avram_raluca-ioana@d.umfiasi.ro (R.A.); buzuleac.ana-maria@d.umfiasi.ro (A.-M.B.); cojocariu.salloum@umfiasi.ro (C.C.); carol.stanciu@umfiasi.ro (C.S.); anca.trifan@umfiasi.ro (A.T.); ana.singeap@umfiasi.ro (A.-M.S.); 2 Institute of Gastroenterology and Hepatology, “St. Spiridon” Emergency County Hospital, Bd. Independentei No. 1, 700111 Iasi, Romania; 3 Department of Internal Medicine and Gastroenterology, “Carol Davila” University of Medicine and Pharmacy, 020021 Bucharest, Romania; 4 Department of Internal Medicine and Gastroenterology, University Emergency Hospital, 050098 Bucharest, Romania

**Keywords:** spontaneous bacterial peritonitis, prophylaxis, fluoroquinolones, multidrug-resistant organisms, norfloxacin, rifaximin

## Abstract

Spontaneous bacterial peritonitis (SBP) is the most common bacterial infection in cirrhotic patients. Historically, the bacterial spectrum was dominated by Gram-negative bacteria. However, recent studies showed that fluoroquinolone (FQ)-based prophylaxis promotes the intestinal overgrowth of Gram-positive bacteria and contributes to the selection of quinolone-resistant Gram-negative bacteria, increasing multidrug-resistant (MDR) organism infections. FQ resistance rates reach up to nearly one-third in community-acquired cases and 50% in hospital-acquired cases, raising concerns about FQ efficacy. Moreover, rare but serious side effects further limit FQ use. Predictive factors of FQ treatment failure have been identified, guiding management strategies. Rifaximin has emerged as a promising alternative for SBP prophylaxis, with encouraging results. This review aims to explore the shifting role of FQ-based SBP prophylaxis, focusing on the emerging concerns, side effects, and alternative strategies. While norfloxacin remains a first-line prophylactic in cirrhotic patients with low ascitic protein levels, its efficacy appears to be reduced in those with advanced liver failure or additional risk factors for MDR organisms. In these subgroups, alternative prophylactics, such as trimethoprim–sulfamethoxazole or rifaximin, may be preferable. We propose a risk-stratification approach to guide treatment selection, with further studies needed to refine these criteria.

## 1. Introduction

Infections are a common and severe complication in patients with liver cirrhosis, having a significant impact on morbidity and mortality. They can lead to further decompensation, including variceal bleeding, acute kidney injury–hepatorenal syndrome (AKI-HRS), and hepatic encephalopathy or can precipitate acute-on-chronic liver failure (ACLF) [1]. There are multiple pathophysiological mechanisms involved in infection occurrence, such as bacterial translocation, exacerbated mostly by portal hypertension, gut dysbiosis, cirrhosis-associated immune dysfunction, and genetic factors [2].

**Figure 1:**
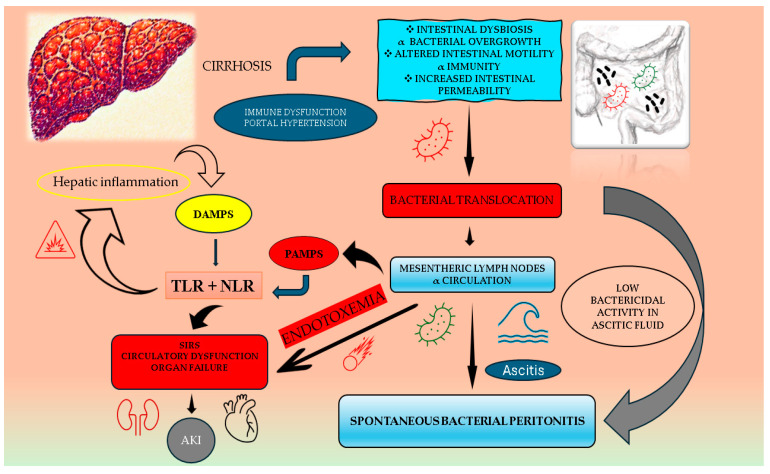
Mechanisms involved in the occurrence of SBP. Intestinal dysbiosis, impaired motility, and increased permeability promote bacterial translocation, which triggers immune dysregulation and systemic inflammation. Pathogen-associated molecular patterns (PAMPs) from translocated bacteria and damage-associated molecular patterns (DAMPs) from injured hepatocytes activate Toll-like receptors (TLRs) and nucleotide-binding oligomerization domain-like receptors (NLRs), further amplifying inflammation and aggravating liver damage. Endotoxemia, a key driver of systemic inflammatory response syndrome (SIRS), is closely linked to hyperdynamic circulation, coagulopathy, acute kidney injury, and multi-organ dysfunction.

Spontaneous bacterial peritonitis (SBP), defined as the infection of ascitic fluid, without an apparent intra-abdominal source [3], is the most common bacterial infection in patients with cirrhosis and ascites (Figure 1), with a prevalence of 10–30% [4]. Mortality following the first episode of SBP ranges from 10% to 50% [5,6]. Often, SBP triggers AKI (Table 1) and ACLF, leading to high short -term mortality rates (Table 2).

Early diagnosis is critical for initiating treatment, which is based on antibiotic therapy and volume expansion with albumin [21]. For patients who survive an initial episode of SBP, the recurrence rate is concerningly high at 69%, with a one-year mortality rate of 62% [22].

Hence, the high rates of recurrence and mortality justify the need for long-term prophylaxis. According to clinical practice guidelines, antibiotic prophylaxis should be given as primary prophylaxis to patients with acute gastrointestinal hemorrhage, to those with risk factors (ascitic fluid protein content lower than 15 g/dL, total bilirubin higher than 3 mg/dL, Child–Pugh score higher than 9, and serum creatinine higher than 1.5 mg/dL or serum sodium lower than 130 mmol/L), and to patients with a history of SBP, as secondary prophylaxis (Table 3) [23,24,25,26].

All patients hospitalized with suspected SBP should undergo an analysis of ascitic fluid to promptly initiate antibiotic therapy if necessary [27]. SBP is typically a monomicrobial infection, most caused by Gram-negative aerobic enteric organisms (e.g., *Escherichia coli*, *Klebsiella pneumoniae)* or Gram-positive aerobic organisms (e.g., *Streptococcus* spp., *Enterococcus* spp.). The diagnosis is based on an ascitic fluid polymorphonuclear cell (PMN) count greater than or equal to 250 cells/microliter, with or without a positive ascitic fluid culture [28]. For these cases, empirical antibiotic therapy, generally with intravenous third-generation cephalosporin, should be initiated promptly. It is crucial to stratify SBP as nosocomial, community acquired, or healthcare-associated to tailor antibiotic regimens, reduce mortality, and develop improved strategies for patient outcomes [29,30].

Fluoroquinolone (FQ)-based antibiotic prophylaxis has been widely used and considered effective in preventing SBP. FQ are broad-spectrum antibiotics that exert their antibacterial effects by inhibiting two essential enzymes involved in DNA synthesis, DNA gyrase and topoisomerase IV [31]. As a first-line option for SBP prophylaxis, their administration is strongly supported by the current guidelines. Based on their antimicrobial activity, FQs are classified into four generations. The first two generations are less effective against Gram-positive bacteria (GPB), while the latter two have a broader Gram-positive coverage [32].

However, in recent years, the bacterial landscape has shifted, with an increasing prevalence of GPB and with multidrug-resistant (MDR) organisms in cirrhotic patients, accounting for 30–35% of infections [33]. Long-term FQ use has been associated with the intestinal overgrowth of Gram-positive species and the selection of quinolone-resistant Gram-negative bacteria [6]. Moreover, studies have linked FQ treatment to a higher risk of infections with MDR organisms [34].

Recent epidemiological studies have highlighted an increasing incidence of rare but clinically significant adverse effects associated with FQs, such as tendinopathy and tendon rupture, aortic aneurysm rupture, and dysglycemia, further limiting their use [35]. The rising resistance rates, the changing bacterial spectrum of SBP, and the side effects of FQs have led to a reassessment of their role in primary and secondary prophylaxis of SBP.

Given the limitations in FQ use, exploring alternative therapeutic options for SBP prophylaxis has become increasingly important. Agents, such as trimethoprim/sulfamethoxazole (TMP-SMX) and rifaximin, emerged as viable options, depending on the clinical setting.

Our review highlights the evolving concerns regarding FQ-based SBP prophylaxis, including resistance issues, treatment failure predictors, and safety concerns. Additionally, it synthesizes emerging evidence on alternative prophylactic agents, providing insights into potential adjustments in current management strategies.

## 2. Multidrug-Resistant Organisms: A Rising Threat

The effectiveness of FQs in preventing SBP appears to have declined in recent years, likely influenced by the rising prevalence of MDR organisms. These include extended-spectrum β-lactamase (ESBL)-producing GNB, fluroquinolone-resistant (QR) GNB, cefoxitin/methicillin-resistant *Staphylococcus aureus* (MRSA), and vancomycin-resistant *Enterococcus* (VRE). In addition to the rising prevalence of GPB, MDR organisms have substantially impacted the management of SBP [36].

Recent studies indicate FQ-resistance rates ranging from 14–33% in community-acquired cases [37,38,39] to 50%, when hospital-acquired cases are considered [40]. The emergence of resistant strains has become a significant challenge in preventing and managing SBP.

Previous exposure to antibiotics is a well-recognized contributor to the development of drug resistance. A recent retrospective study involving 7553 patients who experienced a first episode of SBP, with or without antibiotic prophylaxis, identified *E. coli*, *K. pneumoniae*, and various *Staphylococcus* species as the most common pathogens. Notably, a higher rate of FQ resistance was observed in patients treated with ciprofloxacin compared to those without prophylaxis (34% vs. 14%, *p* < 0.0001). Moreover, the primary prophylaxis for SBP was the only variable associated with Gram-negative resistance [37].

Previous treatment with FQs within 30 days prior to the diagnosis of SBP and a lower SOFA score (assessing sepsis-related organ failure) have been linked to SBP caused by GPB [41]. Additionally, FQ exposure has been demonstrated as a risk factor for developing MRSA infections [34]. The CANONIC study group recently highlighted the escalating prevalence of MDR organisms in culture-positive infections among decompensated cirrhotic patients across Europe [42]. Furthermore, the failure of antibiotic prophylaxis for SBP was correlated with a high prevalence of colonization by MDR organisms, as reported by Mucke et al. in a German prospective observational cohort study [34].

Screening for MDR organisms appears to be a practical tool for identifying patients at risk for SBP caused by MDR pathogens. In a retrospective study of 133 patients diagnosed with SBP, 56.4% had culture-positive SBP, and 16.5% had SBP caused by MDR organisms. Rectal swabs detected MDR organisms in 17 of 22 patients who later developed MDR organism-related SBP, demonstrating time-dependent sensitivities of 77% and 87% at 30- and 90-days post-testing, respectively. The negative predictive values were 83% and 76% for these time intervals [43].

Multidrug resistance is inherently associated with nosocomial SBP. A recent review involving 2302 patients with SBP found that resistance rates to third-generation cephalosporins were higher in nosocomial SBP compared to healthcare-associated and community-acquired SBP. Additionally, the mortality rate was significantly higher in nosocomial SBP compared to both healthcare-associated SBP (RR 1.84, CI 1.43–2.37) and community-acquired SBP (RR 1.69, CI 1.4–1.98). However, there was no significant difference between the mortality rates of healthcare-associated SBP and community-acquired SBP [29]. Another recent systematic review and meta-analysis similarly reported a higher mortality rate for nosocomial SBP compared to community-acquired SBP (OR 2.78, 95% CI 1.87–4.11) [44]. A large retrospective Brazilian study involving 113 cirrhotic patients with monobacterial-culture-positive ascitic fluid found MDR bacteria in 47% of the samples, while resistance to third-generation cephalosporin was observed in 14% of community-acquired SBP cases versus 45% of nosocomial SBP cases. Furthermore, quinolone resistance was absent in community-acquired SBP but reached 49% in nosocomial SBP cases [39]. Given the significant variations in the resistance prevalence and mortality rates between nosocomial and community-acquired SBP, a critical challenge arises in selecting appropriate treatment strategies. Accurate distinction between these types is essential to ensure effective management.

Given the necessity of tailoring empirical antibiotic regimens to specific geographic regions, prospective studies become even more crucial in capturing regional resistance patterns and guiding localized treatment strategies. In a multicenter worldwide prospective study analyzing 354 SBP cases, Piano et al. reported a global MDR organism prevalence of 34%, with notable geographic variations, the highest being in Asia [45]. Additional prospective trials are needed to explore regional resistance patterns further and refine treatment approaches accordingly.

While numerous studies suggest a strong association between prior FQ exposure and increased resistance, some findings question this link. For instance, a prospective study in Greece reported that 20.8% of isolates were MDR, and 10% were extensively drug resistant (XDR). However, the development of MDR/XDR infections was not linked to prior FQ exposure, as few patients had received FQs for SBP prophylaxis [46]. Similarly, a recent retrospective study found that 40% of microbial agents were classified as MDR, while 10% were XDR, yet antibiotic prophylaxis had no effect on the resistance rates of the isolates [47]. These discrepancies suggest that additional prospective research is needed to elucidate the precise conditions under which FQ resistance emerges, considering factors, such as local resistance patterns, patient selection, and prior antibiotic exposure.

Considering the growing burden of MDR pathogens and regional variability in resistance patterns, empirical antibiotic regimens should be regularly reassessed and adjusted accordingly. In high-resistance settings, alternative prophylactic strategies may need to be considered.

## 3. The Etiological Spectrum of Spontaneous Bacterial Peritonitis: A Shift Toward Gram-Positive Organisms

Over recent years, a clear trend has emerged, with a steadily rising prevalence of GPB as etiologic agents in SBP. This shift has significant implications for prophylactic strategies, particularly those centered on FQs, which primarily target GNB.

A comprehensive analysis of global epidemiological data reveals a consistent pattern across different geographic regions and time periods.

In Southern Europe, a retrospective evaluation by Cholongitas et al. [48] observed that the GPB infection rate increased from 25% in patients admitted between 1998 to 1999 to 59.1% in those admitted from 2000 to 2002. Similarly, another Greek retrospective study, including cases from 2008 to 2011, found GPB to be responsible for 55% of SBP cases [49].

This trend is mirrored in Asia, where Guo et al. demonstrated an increasing GPB prevalence from 48% (2011–2013) to 53.4% (2014–2016) (53.4% vs. 48%) [50]. Another Chinese study spanning 1996–2010 showed a dramatic shift, with BGN decreasing from 72.9% to a minority of cases, as GBP (particularly *S. aureus*) became the predominant pathogens [51]. The rising prevalence of GPB in SBP was further highlighted in a retrospective study by Jingjing et al. [50], which evaluated 247 cirrhotic patients with SBP. Among them, 139 patients had positive ascitic fluid cultures; 47.5% of isolated strains were GBP, while 52.5% were GNB. Within the GNB group, *Escherichia coli* was the most common isolate (39.4%), while *Streptococcus* spp. (23.3%) was most prevalent in the GPB group, followed by *Enterococcus* and *Staphylococcus* spp. (38.3%).

In Western Europe, a retrospective German study (2001–2011) found GPB in 53.7% of SBP cases, with *Enterococcus* spp. being the most common [52]. French data from 1996–2001 indicated high proportions of GPB in ascitic fluid (68.3%), with *Enterococcus* spp. (43/125), *Streptococcus* spp. (43/125), and *Staphylococcus aureus* (36/125) as the predominant pathogens [53]. More recent French studies reported GPB in 47.4% to 56.1% of SBP cases [54].

In North Africa, a recent Egyptian cohort cross-sectional study evaluating 72 patients with SBP reported that there were 34 culture-positive patients, from which 27 were GPB and only 7 were GNB [55].

North and South American data further confirm this global phenomenon. In a retrospective study conducted in the United States, GPB were isolated in up to 80% of SBP cases [56], while a Brazilian retrospective study comprising 63 cirrhotic patients over a 5-year period reported a high prevalence of GPB, with Streptococcus spp. accounting for 23.8% (15/63) and *S. aureus* for 7.9% (5/63) [57].

GPB appear to be closely associated with nosocomial infections. A comparative study of 95 patients with cirrhosis and SBP reported that GPB were isolated exclusively from patients with nosocomial infections [58]. However, a recent retrospective study involving 63 cirrhotic patients with culture-positive SBP found that the prevalence of GPB was comparable between nosocomial and non-nosocomial infections (45% vs. 42.2%; *p* = 0.835), though MDR infections were significantly more frequent in the nosocomial group (50% vs. 23.8%; *p* = 0.046) [59]. This suggests that while healthcare exposure remains an important risk factor, the shift toward GPB is occurring broadly, affecting community-acquired infections as well.

The increasing prevalence of GPB in SBP infections across diverse patient populations over the last decades has been supported by additional studies [60,61,62,63]. Other reports have identified GPB in nearly half of culture-positive SBP cases [38,46,64,65,66,67].

All these findings conclude that the shift toward GPB is a consistent global trend that has occurred over the past two decades across different continents and healthcare systems. While regional variations exist, the overall direction of change remains remarkably consistent. Additionally, *Enterococcus* spp., *Streptococcus* spp., and *Staphylococcus aureus* are the most isolated GPB worldwide.

Several interconnected mechanisms likely contribute to the increasing prevalence of GPB in SBP: selective pressure from FQ prophylaxis, changes in the intestinal microbiome composition in advanced liver disease, use of proton pump inhibitors (commonly prescribed to cirrhotic patients), which reduce gastric acid and allow for overgrowth of oral-type flora, healthcare-associated exposure, and changes in host immune defenses, including impairments in those involving the gut–liver axis.

The etiological shift toward GPB presents several diagnostic challenges. Firstly, culture-positivity rates differ, being lower for GPB in standard culture media compared to GNB [68]. Due to the low colony count, many cultures may yield negative results. Additionally, some pathogenic bacteria may enter a viable but non-culturable (VBNC) state, characterized by a loss of culturability, despite maintaining measurable metabolic activity, and the potential for resuscitation under specific conditions [69]. This distinguishes VBNC cells from uncultured bacteria, which cannot grow due to unsuitable conditions, and from persister cells, which remain viable but non-growing under stress, by retaining measurable metabolic activity and the ability to produce virulence factors, unlike dormant cells, which exhibit no detectable metabolic activity [69]. At the same time, traditional inflammatory markers may perform differently in GBP versus GNB infections. For instance, procalcitonin levels tend to be higher in GNB sepsis compared to GPB sepsis [70], potentially affecting early recognition. Additionally, the standard empirical treatment with third-generation cephalosporins may show delayed or suboptimal responses in GBP–SBP, especially with *Enterococcus* spp., intrinsically resistant to cephalosporins [71]. To address these challenges, clinicians should consider using enriched culture media and longer incubation periods when GPB are suspected, performing molecular diagnostic techniques when available, maintaining a high index of suspicion for GBP–SBP, and considering early coverage for GPB in empirical regimens for high-risk patients.

The shift in bacterial ecology also has implications for fungal infections. Spontaneous fungal peritonitis (SFP), while less common than bacterial spontaneous peritonitis, represents a severe complication with high mortality (56–90%) [72]. The diagnosis requires a positive fungal culture and a PMN count of ≥250 cells/microliter in ascitic fluid [73]. Prolonged antibiotic prophylaxis may create conditions favorable for fungal overgrowth by suppressing competing bacteria. Studies report SFP prevalence ranging from 0 to 7.2% among spontaneous peritonitis cases, with *Candida* spp. (particularly *C. albicans*) being the most common isolates [74,75]. The delayed diagnosis due to slow fungal cultures, combined with resistance to standard empirical regimens, contributes to poor outcomes in SFP. This highlights the need for balanced prophylactic strategies that consider the entire microbial spectrum, including potential for fungal infection in long-term prophylaxis settings.

Overall, this shifting etiological spectrum necessitates a reevaluation of current prophylactic approaches and consideration of alternatives that provide broader coverage or targeted strategies based on individual risk assessments.

## 4. Disease-Related Factors Impacting Standard SBP Prophylaxis

In patients with advanced cirrhosis (Child–Pugh class C), norfloxacin showed limited efficacy compared to the placebo, as evaluated by Moreau et al. [76]. A total of 291 patients were randomly assigned to receive either norfloxacin (400 mg daily for 6 months) or a placebo. Fewer than 5% of the patients had a prior episode of SBP, and none had previously been treated with FQs. Patients were followed monthly for the first 6 months and then again at 9 and 12 months. The 6-month mortality rates were similar between the two groups: 14.8% (95% CI, 9.3–21.6) in the norfloxacin group versus 19.7% (95% CI, 13.5–26.8) in the placebo group (*p* = 0.21). Additionally, the incidence of SBP and other infections caused by MDR organisms did not differ significantly between the groups. A subsequent systematic review further suggested that the benefit of norfloxacin decreases with increasing serum bilirubin (*p* = 0.012) and ascites protein (*p* = 0.021) levels [77], raising concerns regarding its efficacy in advanced cirrhosis.

Given these limitations, alternative prophylactic strategies have been explored for Child–Pugh C cirrhosis, particularly in patients at a high risk of MDR infections.

Trimethoprim–sulfamethoxazole has demonstrated comparable efficacy to norfloxacin in preventing SBP, with potential advantages in covering GPB [78]. Observational studies suggest that TMP–SMX may be particularly useful in settings with high fluoroquinolone resistance, although prospective trials in Child–Pugh C patients remain limited [79]. Another promising alternative is rifaximin, a gut-selective antibiotic that reduces bacterial translocation and endotoxemia. Some studies suggest that rifaximin lowers the SBP risk, particularly in cirrhotic patients with concomitant hepatic encephalopathy, though direct comparisons with fluoroquinolones remain sparse [79].

Beyond prophylaxis, identifying predictors of SBP recurrence is crucial for refining surveillance and treatment strategies. Nearly 40 years ago, Tito et al. demonstrated that low ascitic protein levels independently predicted SBP recurrence [80]. More recent studies have identified additional risk factors, including bilirubin levels higher than 1 mg/dL, age more than 55 years, a urinary tract infection history, albumin levels lower than 28.5 g/L, renal failure, MELD score higher than 14, and hepatic encephalopathy [81,82,83]. Also, positive ascitic cultures and endoscopic hypertensive signs have also been linked to SBP recurrence [84]. Emerging evidence suggests that ascites calprotectin may serve as a potential marker for SBP diagnosis and risk stratification [85,86]. Calprotectin, a neutrophil-derived protein involved in innate immunity, has been investigated for its role in distinguishing infected from non-infected ascitic fluid. A cross-sectional study on 50 patients showed that the serum–ascites albumin gradient and ascitic calprotectin levels were significantly higher in those with SBP. Using a calprotectin cut-off of 5.045, the sensitivity and specificity for detecting SBP were 89% and 86%, respectively [87]. While these findings are promising, calprotectin’s clinical application remains under investigation. Prospective validation in larger cohorts is needed before it can be integrated into routine SBP-diagnostic algorithms.

Identifying predictive factors for SBP recurrence in cirrhotic patients is crucial for enhancing surveillance and improving patient outcomes, as standard prophylaxis might be ineffective in these cases.

While norfloxacin remains a first-line prophylactic in cirrhotic patients with low ascitic protein levels, its efficacy appears to be reduced in Child–Pugh C cirrhosis and in patients with prior fluoroquinolone exposure or a high MDR risk. Alternative prophylactics, such as TMP–SMX or rifaximin, may be preferable in these subgroups, though further studies are needed to refine the treatment-selection criteria.

Based on the evidence reviewed, we propose, in the era of increasing antimicrobial resistance, a risk-stratification approach (Table 4).

## 5. Adverse Events as Additional Limitations

Fluoroquinolones are associated with several adverse events, which pose additional risks for cirrhotic patients, potentially limiting their use as prophylactic agents. Their systemic administration can lead to adverse events across multiple body systems, with an incidence ranging from 1 to 10 per 10,000 patients. Since there is no proven treatment for these side effects, FQs may cause impairing, prolonged, and potentially irreversible reactions. Therefore, current guidelines recommend their use only when alternative antibiotics are inappropriate [88].

Initially, FQs were thought to present a low risk of *Clostridioides difficile* infection compared to other antibiotics. However, subsequent reports have shown that FQs may precipitate *Clostridioides difficile*-associated diarrhea (CDAD), which increases the number of hospitalizations and worsens patients’ outcome. A review highlighted that CDAD was linked to prolonged hospital stays, adding significant economic burdens [89].

Among FQs, ciprofloxacin is most frequently associated with hepatotoxicity, while levofloxacin and moxifloxacin are less implicated. Certain FQs, such temafloxacin, trovafloxacin, and gatifloxacin, have been withdrawn from the market due to hepatotoxicity [90]. These drugs can alter the expression of genes involved in mitochondrial damage, RNA processing, transcription, and inflammatory processes, leading to hepatotoxicity [91]. Recently data from the FDA Adverse Event Reporting System underscore the association of ciprofloxacin with acute liver failure, which occurs more frequently than with levofloxacin, moxifloxacin, and ofloxacin [92].

Fluroquinolones are also linked to serious cardiac adverse events, including torsade de pointes, due to QT prolongation. Due to their severe cardiotoxicity, sparfloxacin and grepafloxacin have been withdrawn from clinical use. Moxifloxacin, ciprofloxacin, and levofloxacin should be avoided in patients with risk factors for such severe adverse events, based on current data [93].

Other significant adverse events limiting the use of FQs include metabolic side effects, such as dysglycemia (including hypoglycemia and hyperglycemia [94], neuropsychiatric events (with an incidence ranging from 1% to 4.4%, encompassing a broad spectrum of manifestations including encephalopathy, suicidal depression, seizures, psychosis, and mania) [95], and musculoskeletal issues, such as tendinitis, tendon rupture, peripheral neuropathy, and the exacerbation of myasthenia gravis [96].

Given the concerns regarding FQs, alternative antibiotics for SBP prophylaxis, such as rifaximin and TMP–SMX, are increasingly considered. However, after widespread use in SBP, resistance to TMP–SMX and rifaximin may also emerge [97], highlighting the potential for similar resistance issues as seen with fluoroquinolones.

## 6. Alternative Therapeutic Agents

Fluoroquinolones continue to be a cornerstone in SBP prophylaxis, with strong endorsement from major clinical guidelines for both primary and secondary prevention. However, their limitations underscore the necessity for alternative antibiotics, which have been evaluated for their potential to offer comparable efficacy while overcoming these challenges.

Trimethoprim/sulfamethoxazole, a commonly used synergistic combination, works by inhibiting bacterial folic acid synthesis in a dual manner: trimethoprim blocks dihydrofolate reductase, while sulfamethoxazole inhibits dihydropteroate synthase, both key enzymes in the folate pathway [98]. Active against a variety of Gram-positive and Gram-negative bacteria, TMP–SMX has shown comparable efficacy to norfloxacin, as demonstrated by Lontos et al. in a prospective study involving 80 patients treated for primary prophylaxis. Over one year, the incidence of SBP was similar in both groups, with two patients in each group developing SBP (*p =* 0.60) [99]. Similar results were observed in a retrospective study of 69 patients followed for three years, where the prevalence of SBP was comparable between those who administered norfloxacin and those treated TMP–SMX as primary prophylaxis [100]. However, a recent meta-analysis comprising thirteen randomized control trials (1742 patients) evaluating the efficacy of different antibiotics for SBP prophylaxis reported a superior efficacy for TMP–SMX compared to norfloxacin [101]. Regarding resistance, TMP–SMX had a higher resistance rate compared to FQs in a study encompassing 86 cirrhotic patients diagnosed with SBP (19.4% versus 16.1%) [22]. Nevertheless, recent reports indicate that TMP–SMX maintains high sensitivity against *Streptococcus pneumoniae* (100%) and *Proteus* spp. (83.3%) in culture-positive SBP cases, further supporting its continued efficacy [102].

Addressing the increasing recurrence of SBP may involve changing the prophylactic agent. A recent study on recurrent SBP patients found that switching from first-line prophylactic agents to alternatives (cefdinir or trimethoprim/sulfamethoxazole) significantly reduced recurrence rates and the 6-month mortality. Of the 53 patients studied, those who switched had a lower recurrence rate (52% vs. 100%) and reduced 6-month mortality (24% vs. 57.1%). Additionally, patients in the switched group required less intensive care during subsequent admissions (12% vs. 46.4%) [103]. These results highlight the importance of adjusting prophylaxis strategies when fluoroquinolone treatment fails or is poorly tolerated.

Rifaximin is a promising alternative to norfloxacin for SBP prophylaxis, as many studies have compared their efficacy. It is an oral, non-resorbable broad-spectrum antibiotic that functions by inhibiting bacterial RNA synthesis through binding to the beta subunit of bacterial RNA polymerase, thus preventing transcription and bacterial proliferation in the gut [104]. Rifaximin’s ability to prevent bacterial translocation and overgrowth makes it a valuable option for SBP prevention. Cheng et al. [105] conducted a meta-analysis of 13 studies involving 2207 patients, showing that rifaximin was associated with fewer SBP episodes compared to norfloxacin (OR = 0.39, 95% CI: 0.25–0.62, *p* < 0.001). The rifaximin group also had lower mortality (OR = 0.55, 95% CI: 0.34–0.92, *p* = 0.02) and fewer adverse effects (OR = 0.36, 95% CI: 0.22–0.59, *p* < 0.001). Similarly, Song et al. [106] found a decrease in SBP occurrence with rifaximin compared to norfloxacin in both primary prophylaxis (RR: 1.41; 95% CI: 0.96, 2.06; *p*  =  0.08) and secondary prophylaxis (RR: 4.59; 95% CI: 2.02, 10.43; *p*  =  0.0003). Further studies have also shown promising results for rifaximin in reducing SBP recurrence. Elfert et al. [107] conducted a randomized controlled trial with 262 patients, showing a lower SBP recurrence rate in the rifaximin group (3.88%) compared to the norfloxacin group (14.13%, *p* = 0.04) after 6 months of secondary prophylaxis. Similarly, Shamseya et al. [108] conducted a smaller study (*n* = 86) over one year and found a lower SBP occurrence in the rifaximin group (4.7% vs. 14%), though the result was not statistically significant (*p* = 0.265). Lastly, Praharaj et al. [109] followed 142 patients receiving either norfloxacin or rifaximin for primary or secondary prophylaxis and found similar rates of the first episode of SBP between the two groups (14.3% vs. 24.3%, *p* = 0.5) but significantly fewer SBP recurrences in the rifaximin group (7% vs. 39% *p* = 0.004).

Combining rifaximin with norfloxacin appears to be a promising approach. Menshawy et al. [110] conducted a meta-analysis that included six studies with 973 patients receiving either combination therapy or norfloxacin alone. The combination group had a significant lower incidence of SBP (RR 0.58, 95% CI [0.37, 0.92], *p* = 0.02) and hepatic encephalopathy (RR 0.38, 95% CI [0.17, 0.84], *p* = 0.02), compared to those on norfloxacin alone. This strategy could provide broader antimicrobial coverage and reduce the likelihood of resistance development, especially in settings where single-agent therapy may be less effective over time.

Rifaximin’s potential benefits extend beyond the reduction of hepatic encephalopathy episodes, as it is also capable of lowering the incidence of SBP, reducing the occurrence of variceal bleeding, and decreasing all-cause hospital admissions in patients on the liver transplant waiting list, as demonstrated by Salehi et al., in a retrospective study [111]. Consequently, it represents a promising option for comprehensive management, potentially alleviating the burden of multiple liver-related complications and optimizing patient outcomes, particularly in those awaiting liver transplantation.

However, while alternative agents offer advantages, there are concerns about the potential development of resistance over time. TMP–SMX, although effective, may face increasing resistance in certain bacterial strains with prolonged use, which could limit its efficacy in the long term. Similarly, rifaximin, while promising due to its non-absorbable nature and broad-spectrum action, could still face challenges with emerging resistance. These risks underline the importance of the continuous monitoring of resistance patterns and highlight the potential need for combination therapies to mitigate the development of resistance and maintain effective prophylaxis for SBP.

At the same time, the inability to culture certain microorganisms may hinder our understanding of the full spectrum of bacteria involved in SBP infections and may have implications for treatment strategies [112]. This highlights the limitations of standard culturing techniques and underscores the need for alternative therapeutic agents that might offer broader coverage and a more comprehensive approach to managing SBP, considering both culturable bacteria and those in a viable but non-culturable state, as well as truly non-culturable pathogens.

While alternative therapeutic agents and their combinations offer promising solutions for SBP prophylaxis and recurrence prevention, the economic burden associated with SBP, and its complications cannot be overlooked. Preventing SBP in high-risk cirrhotic patients through prophylactic strategies could lead to substantial savings in terms of hospitalization costs, intensive care unit admissions, and long-term management. However, the rise in resistance rates could influence the overall cost-effectiveness, as resistant infections may require more expensive alternative antibiotics. The cost-effectiveness of FQs and alternative agents remains a topic for further evaluation, such as targeted antibiotic use and the implementation of resistance surveillance programs that could help mitigate the economic impact of MDR organisms on healthcare systems.

## 7. Conclusions

The landscape of SBP prophylaxis is rapidly evolving, presenting significant challenges for clinicians in adapting to the shifting bacterial spectrum, the increasing prevalence of MDR multidrug-resistant organisms, and the growing concerns over the adverse effects of FQs. While FQs have long been a cornerstone in the prevention of SBP, recent evidence suggests that their efficacy is diminishing due to the rise of quinolone-resistant Gram-negative bacteria and the unintended promotion of Gram-positive bacterial overgrowth. These developments, coupled with the increasing rate of rare yet serious side effects associated with FQs, underscore the need for a re-evaluation of their role in SBP prophylaxis.

Considering these challenges, it is crucial to identify and adopt alternative prophylactic strategies that not only address the changing microbiological landscape but also minimize the risk of resistance and adverse outcomes. Rifaximin, among other emerging therapeutic agents, has shown promising results in clinical studies, offering a potential alternative with fewer side effects and a more targeted action within the gastrointestinal tract. However, further research is needed to confirm the long-term efficacy and safety of these alternatives.

Given the evolving nature of SBP prophylaxis, clinicians must carefully consider patient-specific factors when choosing antibiotics, including the liver function, comorbidities, and local resistance patterns. The decision to use fluoroquinolones should be tailored to the individual, weighing the benefits against potential risks. Moreover, the integration of future diagnostic innovations, such as biomarkers for risk stratification, may help in the early detection of SBP and guide more personalized, targeted prophylaxis strategies.

Ultimately, the goal should be to tailor SBP prophylaxis to the individual patient’s risk factors, including their underlying liver condition and the prevalence of resistant organisms in the community. As our understanding of SBP pathogenesis and resistance mechanisms continues to grow, so too should our strategies for preventing this serious complication in cirrhotic patients. Continued surveillance, rigorous clinical trials, and a multidisciplinary approach will be essential in optimizing treatment and improving patient outcomes in the face of a rapidly changing medical landscape.

## Figures and Tables

**Table 1:** Incidence of AKI among patients with SBP.

Study, Year	Number of Patients with SBP	Incidence of AKI
Hadi et al. [7], 2024	212	45.7%
Devani et al. [8], 2019	115	46.7%
Hung et al. [9], 2012	2592	5.6%
Wakani et al. [10], 2019	147	27.2%
Sohn et al. [11], 2020	157	42%
Devani et al. [8], 2019	115,359	46.7%
Serper et al. [12], 2025	4330	65.1%
Shah et al. [13], 2023	168	50%

**Table 2:** Short-term mortality rates in cirrhotic patients with SBP.

Study, Year	Number of Patients with SBP (*n*)	Mortality Rate %, Timeframe
Sort et al. [14], 1999	126	43%, in-hospital
Niu et al. [15], 2018	88,167	17.6%, in-hospital
Devani et al. [8], 2019	115,359	16.1%, in-hospital
Serper et al. [12], 2025	4330	15.5%, in-hospital
Hassan et al. [16], 2023	223	27.4%, in-hospital
Lee et al. [17], 2023	245	17.1%, in-hospital 36.3%, 30 days
Hung et al. [18], 2024	925	10.8%, 30 days
Zakareya et al. [19], 2022	200	20%, 30 days
Ramesh et al. [20], 2024	142	67%, 30 days

**Table 3:** Guideline-based antibiotic prophylaxis strategies for SBP.

Clinical Setting	Antibiotic	Dosage	Duration
Cirrhosis and acute gastrointestinal bleeding	Ceftriaxone	1 g intravenous daily	5–7 days
	Norfloxacin	400 mg per os twice daily	
Primary prophylaxis: cirrhosis, low (<1.5 g/dL) total protein in ascitic fluid, advanced liver disease or renal dysfunction	Norfloxacin	400 mg per os daily	Indefinite if ascites is present
	Ciprofloxacin	500 mg per os daily	
	Sulfamethoxazole/Trimethoprim	One double strength tablet per os daily	
Secondary prophylaxis: previous history of SBP	Norfloxacin	400 mg per os daily	Indefinite if ascites is present
	Ciprofloxacin	500 mg per os daily	
	Sulfamethoxazole/Trimethoprim	One double strength tablet per os daily	

**Table 4:** Clinical recommendations for optimizing SBP prophylaxis.

High-Risk Patient Categories	Clinical Context and Additional Risk Factors	Recommendations
Patients with cirrhosis and acute gastrointestinal bleeding	Acute setting requiring hospitalization Increased risk of bacteremia and other infections beyond SBP	Ceftriaxone (1 g/24 h) for up to 7 days if decompensated cirrhosis, already on FQ, or if high local prevalence of FQ-resistance Oral norfloxacin (400 mg b.i.d.) for the rest
Patients with cirrhosis and low (<1.5 g/dL) total protein in ascitic fluid (primary prophylaxis)	Additional risk factors: renal dysfunction, advanced liver failure (Child–Pugh class C) Risk factors for MDR organisms: prior hospitalization, recent invasive procedures, previous antibiotic exposure, local high prevalence of MDR organisms	Standard oral norfloxacin (400 mg daily) Consider TMP–SMX or rotating antibiotic strategies if additional risk factors Perform rectal swab screening: if MDR colonization, individualized prophylaxis based on susceptibility patterns
Patients with previous history of SBP (secondary prophylaxis)	Additional risk factors for recurrence: bilirubin levels > 1 mg/dL, age > 55 years, history of urinary tract infection, serum albumin levels < 28.5 g/dL Risk factors for MDR organisms: prior hospitalization, recent invasive procedures, previous antibiotic exposure, local high prevalence of MDR organisms	Prior SBP caused by non-MDR organisms: standard oral norfloxacin (400 mg daily) Prior SBP caused by MDR organisms: guided prophylaxis based on isolates’ susceptibility Consider rifaximin (550 mg twice daily) as an alternative, particularly if concurrent hepatic encephalopathy Assessment for liver transplantation

## Data Availability

Data are contained within the article.
